# Mammalian Taste Bud Cells Utilize Extragemmal 5-Hydroxy-L-Tryptophan to Biosynthesize the Neurotransmitter Serotonin

**DOI:** 10.3389/fncel.2018.00461

**Published:** 2018-11-26

**Authors:** Hong-Ru Pan, Miao Tian, Jian-Bo Xue, Song-Min Li, Xiao-Cui Luo, Xiao Huang, Zhen-Huang Chen, Liquan Huang

**Affiliations:** ^1^Institute of Cellular and Developmental Biology, College of Life Sciences, Zhejiang University, Hangzhou, China; ^2^Monell Chemical Senses Center, Philadelphia, PA, United States

**Keywords:** neurotransmitter, biosynthesis, tryptophan hydroxylase, gene knockout, transportation, signal transmission

## Abstract

Serotonin or 5-hydroxytryptamine (5-HT) is an important neurotransmitter that is found in mammalian taste buds and can regulate the output of intragemmal signaling networks onto afferent nerve fibers. However, it is unclear how 5-HT is produced, synthesized locally inside taste buds or absorbed from outside sources. In this study, we attempt to address this question by delineating the process of possible 5-HT biosynthesis within taste buds. First, we verified that the rate-limiting enzyme tryptophan hydroxylase (TPH2) responsible for converting L-tryptophan into the intermediate 5-hydroxy-L-tryptophan (5-HTP) is expressed in a subset of type II taste bud cells (TBCs) whereas the enzyme aromatic L-aromatic amino acid decarboxylase (AADC) capable of converting 5-HTP into 5-HT is found in type III TBCs. And abolishment of TPH2 did not affect the production of intragemmal 5-HT or alter TBCs; the mutant mice did not show any changes in behavioral responses to all five primary taste qualities: sweet, umami, bitter, salty, and sour. Then we identified that 5-HTP as well as AADC are abundant in type III TBCs; and application of an AADC inhibitor significantly blocked the production of 5-HT in taste buds. In contrast, administration of an inhibitor on serotonin-reuptake transporters had minimal impact on the 5-HT amount in taste buds, indicating that exogenous 5-HT is not a major source for the intragemmal transmitter. Taken together, our data indicate that intragemmal serotonin is not biosynthesized *de novo* from tryptophan; instead, it is produced by AADC-mediated conversion of 5-HTP absorbed from the plasma and/or nerve fibers into 5-HT. Thus, our results suggest that the overall bodily 5-HTP level in the plasma and nervous system can regulate taste buds’ physiological function, and provide an important molecular mechanism connecting these peripheral taste organs with the circulatory and nervous systems.

## Introduction

Taste sensation is important to animals’ survival, development, and reproduction. Humans are believed to have five basic taste qualities: bitter, sweet, umami, sour, and salty (Chandrashekar et al., 2006). Mammalian taste buds are the peripheral taste organs that consist of about 50–100 slender taste bud cells (TBCs) embedded in the lingual epithelia (Yarmolinsky et al., 2009).

Mature TBCs, based on their differential morphology and function, are generally categorized into three major types: I, II, and III (Chaudhari and Roper, 2010; Bigiani and Prandi, 2011). Type I cells have glia-like morphology and play supportive roles to other TBCs whereas type II cells are receptor cells, sensing sweet, bitter, and umami tastes with G protein-coupled receptors TAS1Rs and TAS2Rs. And the third type cells, type III, are also known as presynaptic cells, expressing ion channel receptors for sour and salty stimuli and making synapses with the afferent nerve fibers. In addition, multiple neurotransmitters and their receptors are also found in different types of TBCs. For example, adenosine triphosphate (ATP) and acetylcholine (ACh) are released by type II cells while γ-aminobutyric acid (GABA), serotonin (5-hydroxytryptamine or 5-HT) and norepinephrine (NE) can be utilized by type III cells. Furthermore, type II cells also express the ATP receptors P2X2, P2Y1, muscarinic ACh receptors, GABA receptors GABA_A,__B_, and serotonin receptor 1-HT1A that are subject to ATP, ACh, GABA, and 5-HT autocrine or paracrine regulation, respectively (Kinnamon and Finger, 2013; Roper, 2013; Chaudhari, 2014). Type III cells instead express NMDA glutamate receptor, ATP receptor P2Y4 that can be activated by intragemmal neurotransmitters (Roper, 2006). The exact intragemmal cell–cell signaling networks and their impact on the output signals onto the afferent nerve fibers, however, remain to be fully understood.

Among these neurotransmitters, 5-HT has been frequently used to label type III cells (Kim and Roper, 1995). However, the origin of 5-HT is still unclear. The biosynthesis of 5-HT requires two enzymes: (1) tryptophan hydroxylase (TPH), which is a rate limiting enzyme converting L-tryptophan into 5-hydroxytryptophan (5-HTP); (2) L-aromatic amino acid decarboxylase (AADC), which catalyzes the conversion of 5-HTP to 5-HT. Two different forms of tryptophan hydroxylase are involved in the biosynthesis of 5-HTP: TPH1 and TPH2 (Walther and Bader, 2003). TPH1 is mainly distributed in a wide variety of non-neuronal cells, such as enterochromaffin cells of the gut, the pineal gland and skin, whereas TPH2 is expressed in neuronal tissue, specifically in the raphe nuclei of the brain (Slominski et al., 2003; Walther and Bader, 2003; Walther et al., 2003). Previous studies have shown that both TPH1 and TPH2 are expressed in TBCs, but TPH2 is more prominent and selective with no expression in the surrounding tissues while TPH1 is expressed at a lower level and also found in the surrounding non-gustatory lingual epithelia (Ortiz-Alvarado et al., 2006; Dvoryanchikov et al., 2007).

5-HT is an important modulator of diverse biological processes in the central and peripheral nervous systems. In taste buds, 5-HT can be released by type III TBCs in response to tastants (Huang et al., 2007). It can act on afferent nerve fibers as a neurotransmitter or on adjacent TBCs as a paracrine. Indeed, two types of 5-HT receptors, 5-HT1A and 5-HT3, have been found in TBCs (Kaya et al., 2004), of which 5-HT3 receptor may be a part of an elaborate intragemmal signaling network and contributed to physical exercise-induced changes in taste sensitivity and preference (Eccles et al., 2005; Huang et al., 2007; Tsuboi et al., 2015). However, the exact source and role of intra-taste bud 5-HT remain to be fully understood.

In this study, we used gene knockout mice and pharmacological agents to inhibit enzymes and transporters, and found that taste buds mostly rely on exogenous 5-HTP to synthesize intragemmal 5-HT, which provided a molecular link between taste bud function and an individual’s overall physiological state, particularly to the abundance of 5-HTP in the plasma and nervous system.

## Materials and Methods

### Animals

*Tph2* knockout mice, a kind gift from Dr. Yi Rao, Peking University, were generated by deleting exon 5 of the tryptophan hydroxylase gene on the background of C57BL/6J (Gutknecht et al., 2008; Liu et al., 2011). Mice were housed in the ventilated cages at the Laboratory Animal Center of Zhejiang University on a 12 h/12 h light/dark cycle. The wild-type, heterozygote and homozygote mice used for experiments were bred from Tph2^+/-^ heterozygote pairs. For PCR genotyping, the primers used were: Tph2-1 GGGCATCTCAGGACGTAGTAG; Tph2-2 GGGCCTGCCGATAGTAACAC; Tph2-3 GCAGCCAGTAGACGTCTCTTAC (Liu et al., 2011). All experimental procedures with mice were approved by the Animal Care and Use Committee of Zhejiang University.

### Reagents

The chemicals and reagents used in the study were purchased from Sigma-Aldrich, Sanggon Biotech (Shanghai, China) and Xiya Reagent (Chengdu, China). L-Tryptophan was first dissolved in 75 mM NaOH as a stock solution (pH 7.4) and then diluted to the working concentrations. The primary and second antibodies are listed in Supplementary Table 1. The sequences of the primer pairs used for RT-qPCR are listed in Supplementary Table 1.

### Immunohistochemistry

For single antibody staining, animals were anesthetized and transcardially perfused before the lingual tissues were dissected out and fixed in ice-cold 4% paraformaldehyde/PBS for 1 h on ice and cryoprotected in 25% sucrose/PBS in 4°C overnight. The tongues were then sliced into 10–16 μm-thick sections using a cryostat. The lingual sections were washed in PBS for three times, 10 min each time, and incubated in the blocking buffer (3% BSA, 0.3% Triton X-100, 2% donkey serum and 0.1% sodium azide in PBS) at room temperature for at least 2 h. The sections were then incubated with a primary antibody in the blocking buffer at 4°C overnight before being incubated with a secondary antibody at room temperature for 1 h.

For co-localization studies, the first few steps were the same as those described above for single antibody staining. However, after the first secondary antibody incubation, the sections were again blocked in the blocking buffer, followed by incubation with the second primary antibody and second secondary antibody. To visualize the nuclei, 4,6-diamidino-2-phenylindole (DAPi) was applied onto the tissue sections, which then were covered and protected by anti-fade mounting medium (Vector labs).

In some experiments, animals were injected intraperitoneally with 5-HTP (5-hydroxy-L-tryptophan, 20 mg/kg body weight; Sigma-Aldrich), L-tryptophan (100 mg/kg body weight, Sangon Biotech) or pargyline (100 mg/kg body weight, Sigma-Aldrich), 1.5 and 2 h before anesthetization, respectively. For *ex vivo* experiments, tongue tissue blocks of 3 mm × 3 mm × 2 mm containing the circumvallate papillae were isolated from the rest of the tongue and incubated in Tyrode’s solution with or without NSD-1015 (10 mg/mL, 1 h in 37°C), followed by Tyrode’s solution containing 5-HTP (2 mg/mL, 1 h in 37°C). The tissues were then processed for immunohistochemistry as described above.

For cell counting, we followed the previously described procedures (Feng et al., 2012, 2014). In brief, to quantify the number of TPH2 expressing cells in taste tissues, two circumvallate sections at the middle ranges of the papillae, separated from each other by at least 40 μm (four 10-μm-thick sections away) to avoid double counting of any cells, were selected for immunohistochemistry from serial sections of each papilla. Fluorescent images were acquired using Zeiss LSM710 or LSM780 confocal microscope and their size and contrast were minimally adjusted using Photoshop CC (Adobe).

### Real-Time Quantitative PCR Experiments

Murine lingual RNA was prepared as described previously (Feng et al., 2012, 2014). Briefly, murine lingual epithelia were isolated by injection of Ca^2+^ -free Tyrode’s solution (140 mM NaCl, 10 mM HEPES, 5 mM KCl, 1 mM MgCl_2_, 10 mM Na pyruvate, 10 mM glucose, and 2 mM EGTA, pH 7.4) containing a mixture of enzymes (2 mg/mL dispase II, 1 mg/mL collagenase A, and 1 mg/mL trypsin inhibitor) between the epithelial layer and its beneath muscle. After 15–20 min incubation at 37°C, the epithelium was peeled off from the rest of the tongue, and the circumvallate and foliate papillae as well as the anterior portion of the epithelium enriched with the fungiform papillae were excised and used for total RNA extraction with the Absolutely RNA Microprep Kit (Agilent). The first-strand cDNA was synthesized from 1 to 2 μg total RNA using the SuperScript^TM^ III Reverse Transcriptase (Invitrogen) following the manufacturer’s instruction. The quantitative real-time PCR reactions were set up using the PCR primers listed on Supplementary Table 1 and iQ^TM^ SYBR^®^Green supermix (Bio-Rad), and performed on a CFX^TM^ Connect Real-Time PCR Detection System (Bio-Rad). Relative gene expression levels were determined based on the 2^-ΔΔCt^ method using the CFX manage software. *Gapdh* was used as an internal reference gene for the analyses. The experiments were repeated three times and the data from these experiments were used for statistical analyses.

### Behavior Experiments

Two-bottle preference tests were conducted as described previously (Gaillard and Stratford, 2016). Series of solutions representing five basic taste qualities were tested from low to high concentrations (mM): sucrose, 10, 20, 40, 80, 160, 320; NaCl, 3, 10, 30, 75, 100, 150, 200, 300; potassium glutamate, 0.1, 0.3, 1, 3, 10, 100; quinine, 0.006, 0.02, 0.06, 0.3, 1, 3; HCl, 0.2, 0.5, 1, 5, 10, 100; citric acid, 0.1, 0.3, 1, 3, 10, 30.

Brief access lickometer assays were performed on a mouse gustometer (Davis MS 160, Dilog Instruments) as described previously (Glendinning et al., 2002). Briefly, mice were under a restricted water and/or food access schedule for the first 3 days to be trained to sample the sipper tube and then tested for their licking responses to tastants at different concentrations as well as water as a control. The following tastants were tested (mM): sucrose, 10, 30, 200, 300, 600, 1000; NaCl, 10, 100, 200, 300, 600, 1000; denatonium, 0.1, 0.3, 1, 3, 10, 30; quinine, 0.01, 0.03, 0.1, 0.3, 1, 3. The tastant/water lick ratios for most tastants except sucrose were used for statistical analyses whereas standardized lick ratios of tastant lick numbers over the maximal licks were used for analyzing sucrose data (Glendinning et al., 2002).

### Statistical Analyses

Data from two-bottle preference tests were reported in the form of percent preference scores as described previously (Nelson et al., 2010) and presented as means ± SD. Comparison of two groups was analyzed by a two-tailed Student’s unpaired *t*-test. Preference scores for both genotypes of mice were analyzed using repeated measures analysis of two-way variance (two-way ANOVA) using Statistica software (StatSoft, Inc.) and graphs were generated using GraphPad Prism (GraphPad Software, Inc.). Statistically significant differences between groups are indicated as *P* < 0.05.

## Results

### Intragemmal 5-HT Precursor 5-HTP Is Found in a Subset of Taste Bud Cells

**FIGURE 1:**
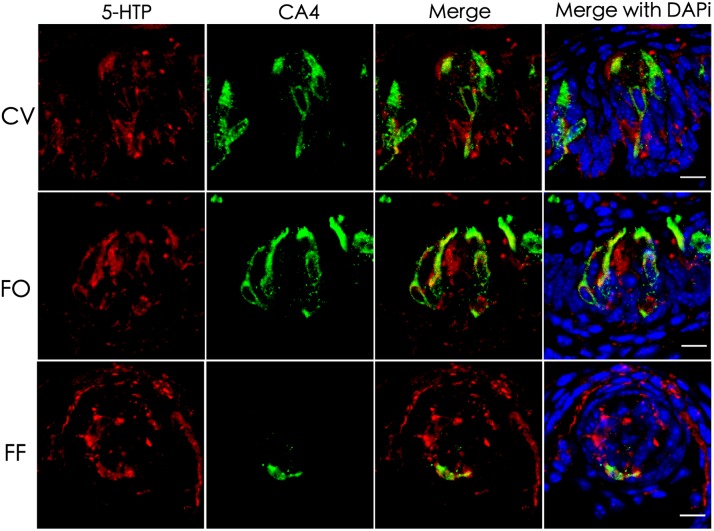
Co-localization of 5-HTP with type III cell marker CA4 in taste buds. Immunostaining of 5-HTP (red) with CA4 (green) on the circumvallate (CV), foliate (FO), and fungiform (FF) sections shows that some 5-HTP-positive cells co-expressed with type III cell marker CA4, indicating that 5-HTP-positive cells were type III taste bud cell (TBCs). Besides, there were also some punctate and streaky 5-HTP immunoreactive signals within taste buds. Z-Stack: 5 μm. Scale bars: 10 μm.

5-HT plays an important role in various tissues and organs (Barnes and Sharp, 1999). In taste buds, 5-HT is detected in type III cells usually following the intraperitoneal injection of its precursor, 5-HTP (5-hydroxytryptophan) (Ren et al., 1999; Yee et al., 2001). To determine the exact source of 5-HT, we performed immunostaining with an antibody against 5-HTP 1 h after the intraperitoneal injection of its precursor L-tryptophan. Our results showed that 5-HTP immunosignals were partially overlapped with type III cell marker carbonic anhydrase (CA4) (Chandrashekar et al., 2009) in the circumvallate (CV), foliate (FO), and fungiform (FF) papillae (Figure 1). In addition, some dotty signals were also found inside taste buds (Figure 1). In contrast, no 5-HTP immunostaining signals were detected in the taste papillae of the negative control mice injected with the vehicle solution (Supplementary Figure 1).

### AADC Is Required for Intragemmal 5-HT Biosynthesis

**FIGURE 2:**
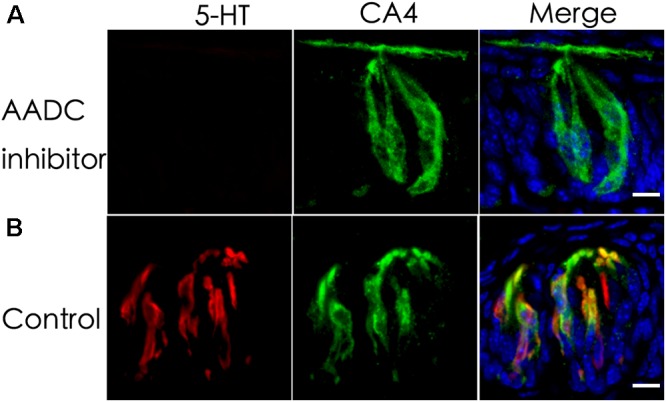
An aromatic L-amino acid decarboxylase (AADC) inhibitor abolishes the generation of 5-HT in TBCs. Mouse lingual tissue blocks containing the circumvallate papilla were excised and briefly cultured *ex vivo* with or without the aromatic AADC inhibitor NSD-1015, which were then processed for immunostaining with antibodies to 5-HT and type III cell marker CA4. Confocal images show that in the presence of both NSD-1015 and the 5-HT’s precursor 5-HTP, no 5-HT immunostaining signals were found in TBCs **(A)** whereas in the control experiment without the inhibitor, 5-HT immunostaining (red) was found in TBCs and colocalized with that of type III cells marker CA4 (green) **(B)**. Z-stack: 6 μm. Scale bars: 10 μm.

L-Amino acid decarboxylase is the enzyme found to be in type III TBCs and capable of converting 5-HTP into 5-HT (Nagatsu, 1991; Seta et al., 2007). To determine whether it is required for biosynthesizing 5-HT from 5-HTP present in type III TBCs, we performed *ex vivo* tissue culture experiments using taste buds-containing lingual tissue blocks. As shown in Figure 2, the incubation of taste tissue blocks with the 5-HTP solution in the presence of the AADC inhibitor NSD-1015 (10 mg/mL) abolished the production of 5-HT in the circumvallate taste buds (Figure 2A). In contrast, in the control samples without the inhibitor, strong 5-HT immunostaining signals were observed (Figure 2B). In comparison with Figure 1, the 5-HT signals in the *ex vivo* tissue (Figure 2) were largely restricted to TBCs *per se* with no or little extracellular signals.

### The Serotonin-Reuptake Transporter Inhibitor Does Not Eliminate 5-HT Inside Taste Bud Cells

**FIGURE 3:**
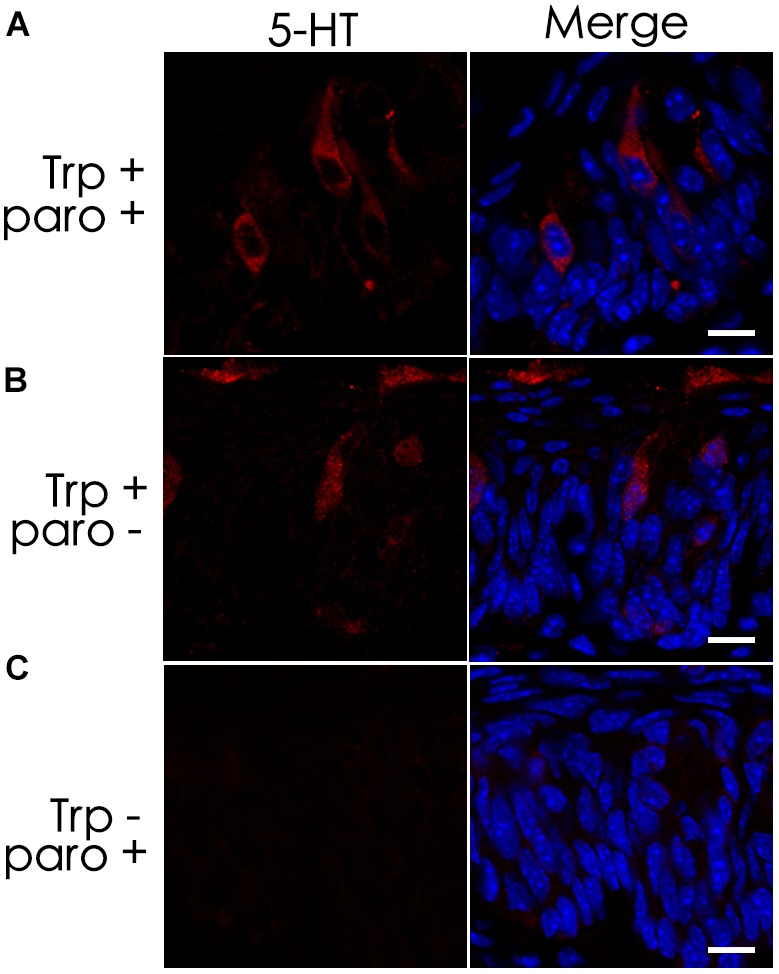
The serotonin-reuptake transporter (SERT) does not contribute to the accumulation of 5-HT in taste buds. Intraperitoneal injection of both the transporter’s inhibitor paroxetine (paro+) and the 5-HT’s precursor L-tryptophan (Trp+) did not eliminate 5-HT’s immunostaining signals **(A)**, which was nearly identical to that of the positive control experiment without the inhibitor (paro-) **(B)**. In the negative control experiment without injection of L-tryptophan (Trp-), no 5-HT immunostaining was found on taste buds **(C)**. Scale bars: 10 μm.

Previous studies have shown the expression of the serotonin-reuptake transporter (SERT or 5-HTT) in type III cells (Ren et al., 1999). To determine whether the SERT contributes to the accumulation of 5-HT in TBCs by its reuptake from the extracellular space, we administered a selective SERT inhibitor, paroxetine, via intraperitoneal injection (Mathes and Spector, 2011; Blier and El Mansari, 2013; Mathes et al., 2013). The results showed that intraperitoneal injection of both paroxetine and the 5-HT precursor L-tryptophan (Trp) did not diminish 5-HT immunostaining signals in a subset of circumvallate TBCs (Figures 3A,B). To exclude the possibility that the 5-HT antibody might non-specifically bind to some other antigens, we also carried out a control experiment without Trp injection, and no 5-HT immunostaining signals were found in taste buds (Figure 3C).

### Tryptophan Hydroxylase 2 (TPH2) Is Expressed in Type II Taste Bud Cells

**FIGURE 4:**
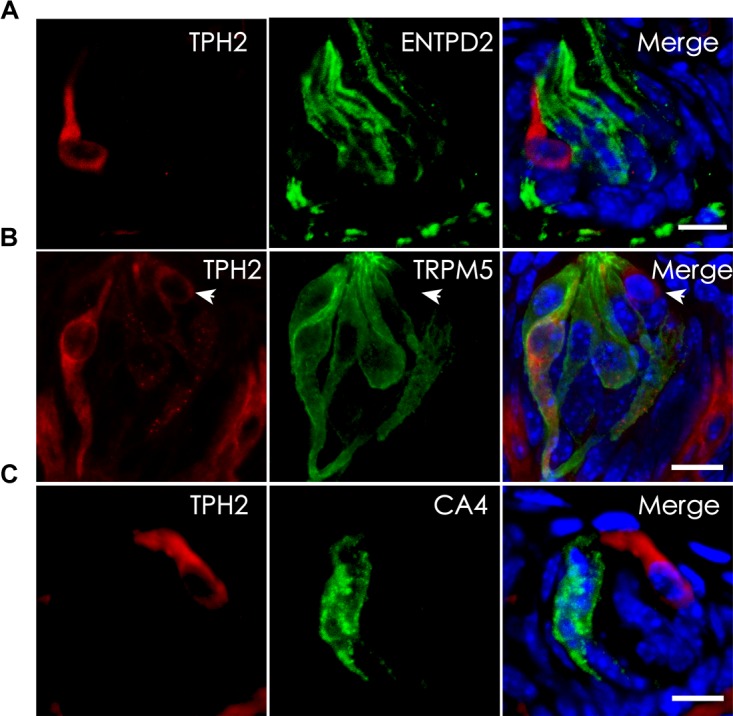
Localization of TPH2 proteins to a subset of type II TBCs. **(A)** Confocal images of double immunostaining with antibodies to TPH2 (red) and the type I cell marker ecto-nucleoside triphosphate diphosphohydrolase 2 (ENTPD2, green) on mouse circumvallate sections show no overlapping. **(B)** Confocal images of double immunostaining with antibodies to TPH2 (red) and the type II cell marker transient receptor potential cation channel subfamily M member 5 (TRPM5, green) indicate the TPH2 expression in a subset of type II taste cells. However, a few TPH2-expressing cells (arrow) did not express TRPM5. **(C)** Confocal images of double immunostaining with antibodies to TPH2 (red) and type III taste cell marker carbonic anhydrase 4 (CA4, green) show no overlapping. Z-Stack: 6 μm. Scale bars: 10 μm.

Tryptophan hydroxylase (TPH) is the rate-limiting enzyme in the 5-HT biosynthesis process that converts L-tryptophan into 5-HTP. Previous studies indicate the presence of two isoenzymes TPH1 and TPH2 while TPH2 is the dominant form in the taste papillae (Ortiz-Alvarado et al., 2006; Dvoryanchikov et al., 2007). To validate THP2 expression and localize its proteins to TBCs, we carried out immunohistochemistry with antibodies against TPH2 as well as TBC type-specific markers: ENTPD2 for type I cells, TRPM5 for type II cells and CA4 for type III cells (Haycock et al., 2002; Bartel et al., 2006; Clapp et al., 2006; Chandrashekar et al., 2009). The immunostaining results with the circumvallate papillae indicated that there was no overlapping between TPH2 immunostaining and that of type I cell marker ENTPD2 (Figure 4A) or between TPH2 and type III marker CA4 (Figure 4C); however, a subset of TRPM5-expressing cells exhibited TPH2 immunoreactivity (Figure 4B). To determine whether the same TPH2 expression pattern also exists in other taste papillae, we carried out immunohistochemical studies with mouse foliate and fungiform papillae as well (Supplementary Figure 2). The results indeed indicated that THP2 had a partial colocalization with TRPM5 to some type II cells whereas no immunostaining signals were detected in the negative control tissue sections for the TPH2 antibody (Supplementary Figure 3). Quantitative analysis of TBCs expressing TPH2 and TRPM5 indicated that out of 760 circumvallate, 336 foliate and 216 fungiform TBCs, about 4.2, 12.4, and 7.4% expressed both TPH2 and TRPM5, respectively, whereas 18.2, 28.9, and 18.1% expressed TRPM5 alone, and 3.7, 3.4, and 3.9% expressed TPH2 alone, respectively (Supplementary Figure 4). About 73.9, 55.3, and 70.6% of circumvallate, foliate and fungiform TBCs, respectively, expressed neither of TPH2 nor TRPM5.

### Nullification of the *Tph2* Gene Did Not Alter Mutant Mice’s Behavioral Responses to Taste Stimuli

Partial overlapping expression pattern between TPH2 and TRPM5 in taste buds prompted us to determine the possible role of TPH2 in taste sensation. We utilized a previously generated *Tph2*-knockout mouse strain (Zhang et al., 2013) and performed two bottle preference tests as well as lickometer tests. The results showed that although animals’ preferences changed over the concentrations of 6 different tastants representing five basic taste qualities, no statistically significant changes were found between *Tph2*-KO (KO) and wild-type control (WT), or between *Tph2*-KO and *Tph2* heterozygotes (HT) (Supplementary Figure 5). Also, no significant differences in licking ratios in response to different tastants were found between *Tph2*-KO and WT mice (Supplementary Figure 6).

### Nullification of the *Tph2* Gene Did Not Alter Taste Buds’ Gene Expression or Cell Composition

To determine whether *Tph2*-KO affects expression of certain genes in mouse taste buds, we performed reverse transcription-quantitative PCR with the primers for the genes encoding some taste signaling proteins. Total RNAs from mouse circumvallate, foliate, and fungiform as well as non-taste lingual epithelium tissue on the back of the tongue from WT and *Tph2*-KO mice were extracted, reverse transcribed into first-strand cDNA and used for qPCR analysis (Wang et al., 2007). The results indicated that there was no significant difference in expression levels of the tested genes, including those for the G protein subunit α-gustducin (*Gnat3*), polycystic kidney disease 2-like 1 protein (*Pkd2l1*), ectonucleoside triphosphate diphosphate hydrolase family member 2 (*Entpd 2*), salty taste receptor ion channel *Scnn1a*, carbonic anhydrase *Car4*, synaptosomal-associated protein 25 (*Snap-25*), *Trpm5*, brain-derived neurotrophic factor *Bdnf* and serotonin transporter *Slc6a4*; and particularly, no significant upregulation of *Tph1* expression was found in *Tph2*-KO sample (Supplementary Figure 7).

**FIGURE 5:**
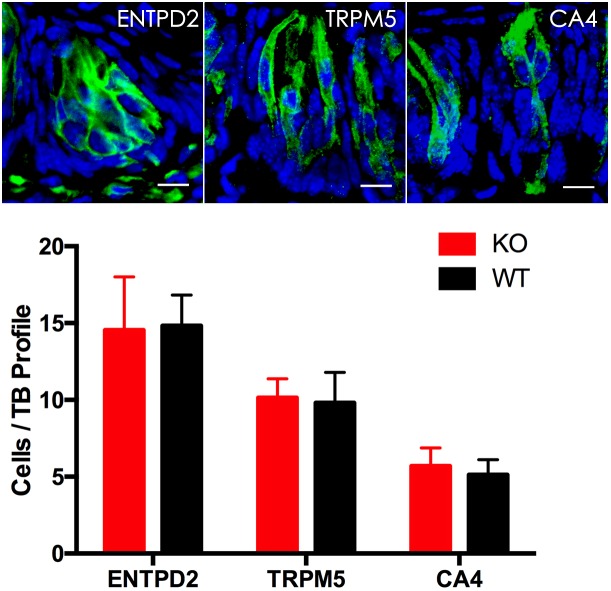
Immunohistochemical studies and statistical analyses of different cell types in *Tph2*-KO versus WT taste buds. Representative confocal images of *Tph2*-knockout taste buds immunostained with antibodies to ENTPD2, TRPM5 and CA4, respectively. The histogram shows that there were no significant differences in the numbers of type I cells (ENTPD2), type II cells (TRPM5), and type III cells (CA4) between *Tph2* knockout and wild-type mice (*t*-test; ENTPD2, *P* = 0.79, TRPM5, *P* = 0.6; CA4, *P* = 0.27). Scale bars: 10 μm.

To determine whether knockout of the *Tph2* gene alters the cell numbers of different types in a taste bud, we performed immunohistochemistry with antibodies to the markers of three TBC types and statistically analyzed the numbers of these cells from *Tph2* -KO versus WT mice. The results showed that *Tph2*-KO taste buds appeared grossly normal in comparison with WT taste buds; one taste bud profile had about 30 cells, of which over 15 were type I cells, 10 type II cells and 4–6 type III cells, consistent with previously reported data (Feng et al., 2014), and no significant differences in the cell numbers of the three types were found between KO and WT taste buds (Figure 5).

### *Tph2*-Knockout Does Not Reduce the Amount of Intragemmal 5-HT

**FIGURE 6:**
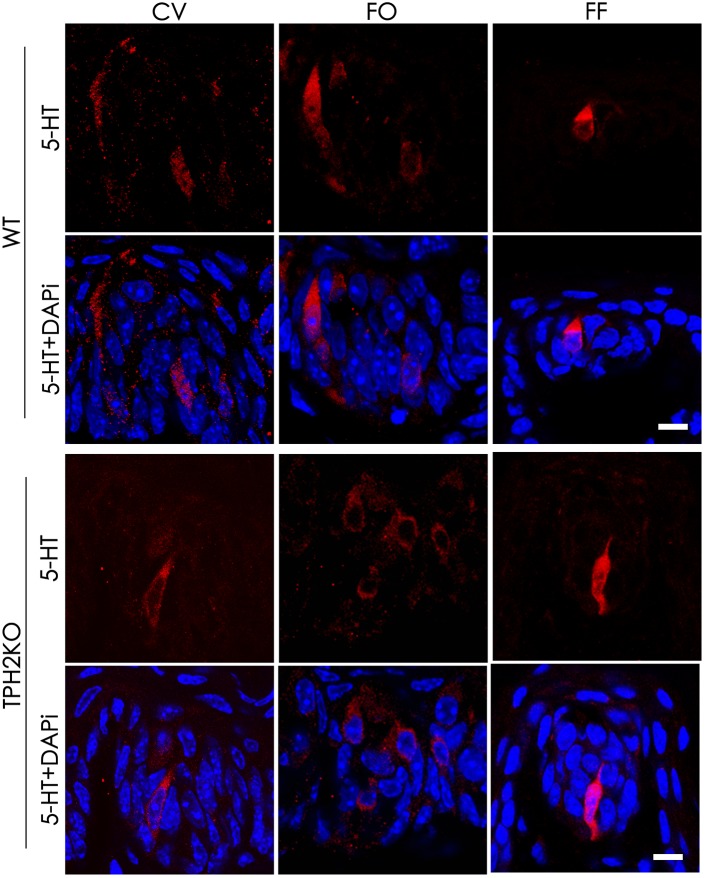
The effect of *Tph2*-KO on the amount of intragemmal 5-HT in mouse taste buds. Confocal images of immunostaining sections of the circumvallate (CV), foliate (FO), and fungiform (FF) papillae with an anti-5-HT antibody. 5-HT-positive cells (red) were found in both WT and *Tph2*-KO taste buds. Z-Stack: 5 μm. Scale bars: 10 μm.

Tryptophan hydroxylase is a rate-limiting enzyme in 5-HT biosynthesis, which converts tryptophan into 5-HTP. In mouse taste buds, *Tph2* is expressed more prominently than the other isoform *Tph1*. To evaluate the contribution of *Tph2* to the production of intragemmal 5-HT, we performed immunohistochemical studies with an anti-5-HT antibody on the circumvallate, foliate and fungiform papillae of both WT and *Tph2*-KO mice. To increase the intragemmal 5-HT detectability, mice were injected intraperitoneally with both the 5-HT’s precursor L-tryptophan and the inhibitor pargyline that blocks 5-HT degradation by L-monoamine oxidase (Kim and Roper, 1995; Yoshihara et al., 2012). The results indicated that 5-HT-positive cells were found in the circumvallate, foliate and fungiform papillae of both WT and *Tph2*-KO mice (Figure 6), and no significant differences in the number of 5-HT-positive cells were found between WT and KO taste buds.

### Minimal *de novo* Synthesis of 5-HT From L-Tryptophan in Taste Buds

**FIGURE 7:**
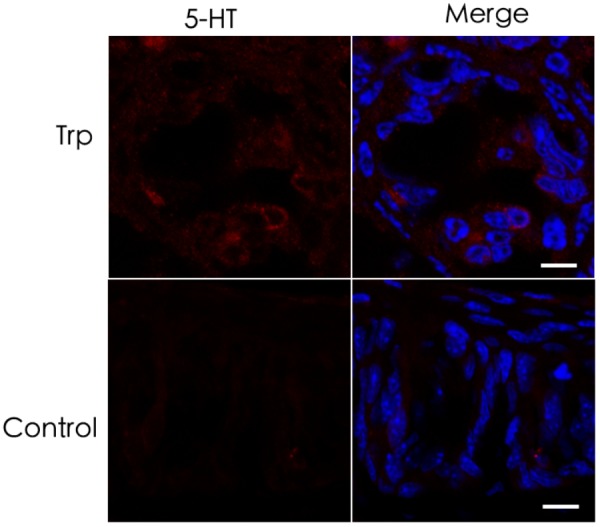
Minimal *de novo* 5-HT synthesis in taste buds. **(top)** *Ex vivo* culture of the circumvallate taste buds in L-tryptophan-containing Tyrode’s solution resulted in hardly detectable 5-HT immunoreactive signals. **(bottom)** *Ex vivo* culture of the circumvallate taste buds in Tyrode’s solution without L-tryptophan produced completely undetectable 5-HT immunoreactivity. Scale bars: 10 μm.

The results showing no reduction of 5-HT in the *Tph2*-KO taste buds prompted us to hypothesize that TBCs may not use L-tryptophan to synthesize 5-HT *de novo* but utilize the intermediary 5-HTP from extragemmal sources. To test this notion, we performed an *ex vivo* tissue culture experiment with isolated circumvallate tissue blocks and incubated them with L-tryptophan (Trp)-containing Tyrode’s solution to deplete most, if not all, 5-HTP from the nerve terminals or blood vessels in the tissue blocks. As shown in Figure 7, only minimal 5-HT immunoreactive signals were observed in the circumvallate cultured *ex vivo*. However, the control experiment without L-tryptophan produced no detectable 5-HT immunoreactivity.

## Discussion

**FIGURE 8:**
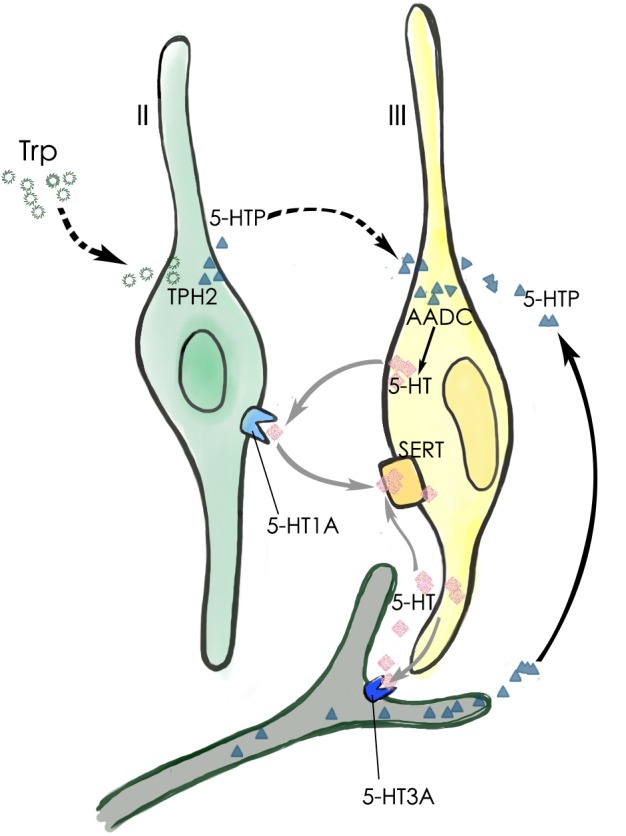
Schematic diagram of 5-HT production and its function in a taste bud. The major 5-HT production pathway (black arrow) is that type III TBCs absorb 5-HT’s precursor 5-HTP from the blood plasma or those released by the afferent and efferent nerve terminals. A minor 5-HTP source (dashed arrows) is that type II cells absorb and utilize L-Trp from the extracellular microenvironment and convert it by TPH2 into 5-HTP, which then diffuses into adjacent type III cells. Once inside type III cells, the enzyme AADC converts it into the neurotransmitter 5-HT, which can act on its receptor 5-HT1A on type II TBCs, 5-HT3A on the afferent nerve fibers, or is reabsorbed back into type III cells by the SERT transporters (gray arrows).

Our data have shown that the neurotransmitter 5-HT present in type III TBCs originates largely from the conversion of 5-HTP by the intracellular aromatic AADC whereas 5-HTP itself is mostly transported into type III cells from extracellular sources such as blood plasma or released from the afferent and efferent nerve terminals (Figure 8) (Takeda, 1977; Kaya et al., 2004; Roper, 2013; Larson et al., 2015). It is also possible that type II TBCs may absorb L-tryptophan from their extracellular microenvironment, convert it into 5-HTP, which diffuses into adjacent type III cells where it is biosynthesized into 5-HT by the AADC (Figure 8). However, its contribution to type III TBCs’ 5-HT production is minimal.

We first confirmed that type III TBCs contain 5-HT’s precursor 5-HTP (Figure 1). However, some 5-HTP immunoreactivity was also found in some other cells or subcellular structures than type III cells, which could be the afferent and efferent nerve terminals or type II cells since neurons can contain 5-HTP while type II cells express TPH2 and are able to convert L-tryptophan into 5-HTP (Figure 4). 5-HTP is then converted into 5-HT by the aromatic AADC, which is expressed in Type III cells. When AADC’s inhibitor NSD-1015 was applied to an *ex vivo* culture of the circumvallate-containing lingual tissue block in the presence of 5-HTP, 5-HT biosynthesis was completely eliminated (Figure 2), suggesting that AADC is the key enzyme to producing intragemmal 5-HT. In addition to the biosynthesis of 5-HT by AADC, another possibility is that 5-HT may also be absorbed by the SERT from the extracellular sources. However, when the SERT inhibitor paroxetine was administrated together with L-tryptophan intraperitoneally, 5-HT immunostaining signals were still abundant in TBCs, which was not non-specific immunosignals since lack of L-tryptophan eliminated the immunostaining (Figure 3). Thus, 5-HT in taste buds was not imported from the extracellular sources; instead, it is mainly biosynthesized from 5-HTP by AADC in TBCs.

To determine whether 5-HTP in taste buds was synthesized *de novo* from L-tryptophan, we first confirmed the expression of the tryptophan hydroxylases that are capable of converting L-tryptophan into 5-HTP. We found that the dominant TPH2 of the two isoforms TPH1 and TPH2 in some of type II TBCs in all the circumvallate, foliate and fungiform papillae (Figure 4 and Supplementary Figure 2), which is consistent with the previous reports (Ortiz-Alvarado et al., 2006; Dvoryanchikov et al., 2007). Quantitative analysis indicated that a small number of type II cells expressed both TPH2 and TRPM5 in the circumvallate, foliate and fungiform taste buds, whereas an even smaller number of cells expressed TPH2 alone, but a much greater number of cells expressed TRPM5 alone (Supplementary Figure 4). Apparently, there were significantly more cells co-expressing both TPH2 and TRPM5 in foliate than in circumvallate whereas cells expressing TRPM5 alone were significantly more prevalent in the foliate than in the other two types of taste papillae. These results suggest that TRPM5-expressing type II TBCs can be further divided into subtypes, for example, TPH2-expressing or not expressing subtypes, which may play different roles in the intragemmal signaling networks. Further studies are warranted to understand the functional differences among these subtypes or the biological significance of their differential distributions among the three types of taste papillae.

To evaluate possible contributions of TPH2 to taste sensation, we performed both two bottle preference tests and brief access lickometer tests with the *Tph2*-knockout mice (Zhang et al., 2013) and WT control. The results showed that the gene knockout did not affect mutant mice’s behavioral responses to sweet (sucrose), bitter (quinine), umami (mono potassium glutamate), salty (NaCl), and sour (HCl, citric acid) tastants (Supplementary Figures 5, 6). Further analyses on the cell compositions and gene expression profiles of the mutant taste buds using immunohistochemistry and qPCR approaches show no alterations in the cell numbers of different types or in expression of *Tph1* and other key genes (Figure 5 and Supplementary Figure 7). Furthermore, *Tph2*-knockout appears not to affect intragemmal 5-HT production following intraperitoneal injection of the precursor L-tryptophan (Figure 6). All these results point to that TPH2 is not essential to the gene expression, TBC composition, intragemmal 5-HT production or behavioral responses of mutant mice to various tastants. However, when isolated taste papillae were incubated with the amino acid L-tryptophan, only residual amount of 5-HT was barely detectable in a subset of TBCs, indicating that TBCs had minimal capability of biosynthesizing 5-HT *de novo* from L-tryptophan.

Taken together, we conclude that: (1) the intragemmal 5-HT is mostly produced by type III TBCs, which employ the intracellular enzyme AADC to biosynthesize 5-HT from 5-HTP, while the contribution of 5-HT reuptake from extracellular space is minimal; (2) the intragemmal 5-HTP is mostly transported from extracellular sources such as blood plasma or afferent/efferent nerve terminals (Schruers et al., 2002; Favale et al., 2003; Amer et al., 2004), whereas the contribution from a subset of type II TBCs that utilize TPH2 to convert L-tryptophan into 5-HTP is secondary; (3) 5-HT released from type III TBCs can act on 5-HT1A receptors on adjacent type II TBCs or 5-HT3A receptors on the nerve fibers, thus affecting intragemmal signaling networks and contributing to the output signals onto the afferent nerves (Figure 8). Since extragemmal 5-HTP is the major substrate for intragemmal 5-HT biosynthesis, 5-HTP levels in blood plasma or nervous system can modulate taste bud signaling activity that leads to taste sensation and perception.

## Ethics Statement

This study was carried out in accordance with the recommendations of Zhejiang University Laboratory Animal Research guidelines. The protocol was approved by the Zhejiang University Institutional Animal Care and Use Committee.

## Author Contributions

LH, H-RP, and MT designed the experiments and interpreted the data. H-RP, MT, J-BX, S-ML, X-CL, XH, and Z-HC performed the experiments. LH and H-RP wrote the paper.

## Conflict of Interest Statement

The authors declare that the research was conducted in the absence of any commercial or financial relationships that could be construed as a potential conflict of interest.
