# Supplementary material for: Mammalian Taste Bud Cells Utilize Extragemmal 5-Hydroxy-L-Tryptophan to Biosynthesize the Neurotransmitter Serotonin
Source: Front Cell Neurosci. 2018 Nov 26;12:461. doi: 10.3389/fncel.2018.00461 (PMC6275321; doi:10.3389/fncel.2018.00461)
Supplement: Supplementary file 2 [file Data_Sheet_1.docx]

Supplementary Material

Mammalian Taste Bud Cells Utilize Extragemmal 5-Hydroxy-L-Tryptophan to Biosynthesize the Neurotransmitter Serotonin

Hong-Ru Pan, Miao Tian, Jian-Bo Xue, Song-Min Li, Xiao-Cui Luo, Xiao Huang, Zhen-Huang Chen, Liquan Huang^*^

**Correspondence:** Dr. Liquan Huang**:** huangliquan@zju.edu.cn


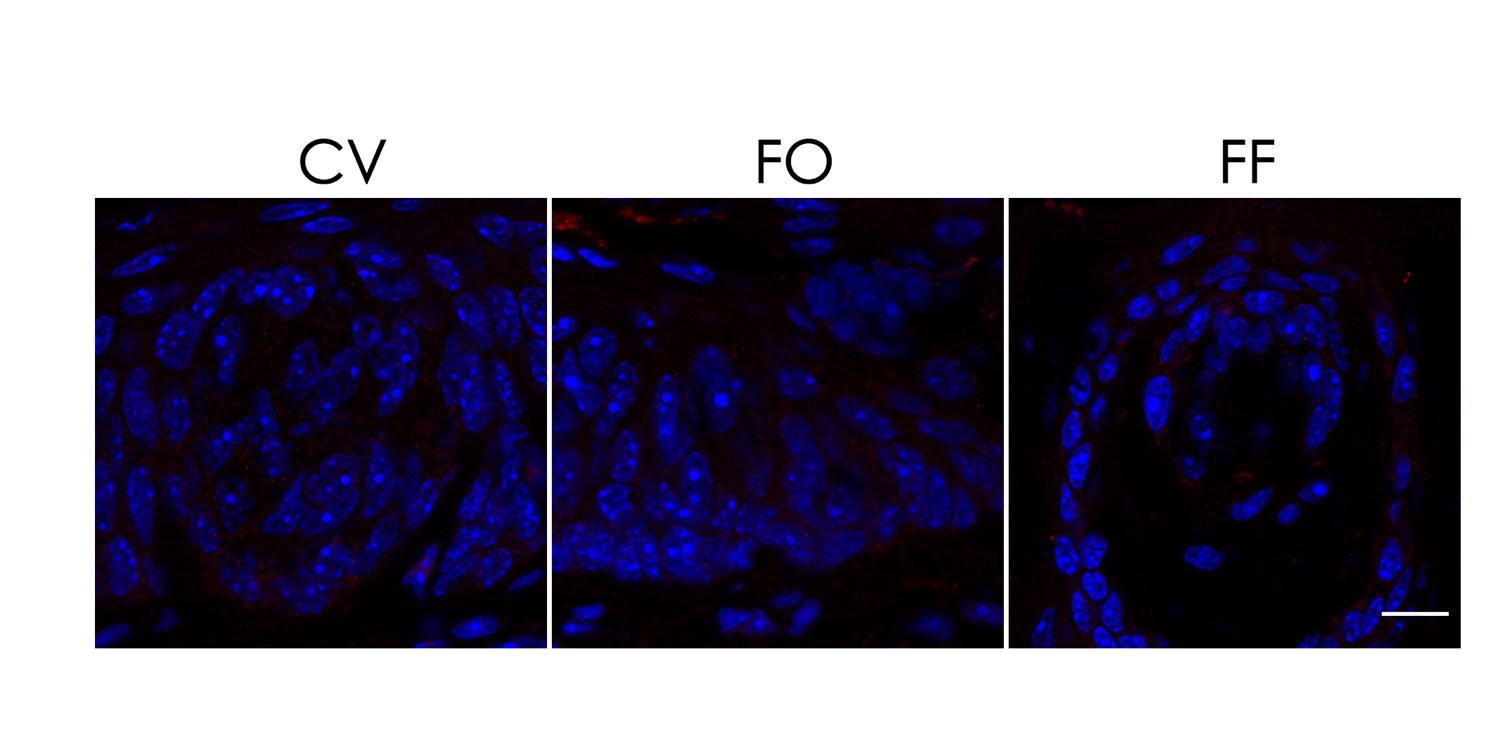


**Supplemental Figure 1 Absence of 5-HTP immunostaining signals in taste buds of the negative control for the anti-5-HTP antibody.** Confocal images show no 5-HTP immunostaining signals in the circumvallate (CV), foliate (FO) and fungiform (FF) papillae without the primary antibody against 5-HTP. Z-Stack: 8 µm. Scale bars: 10 µm


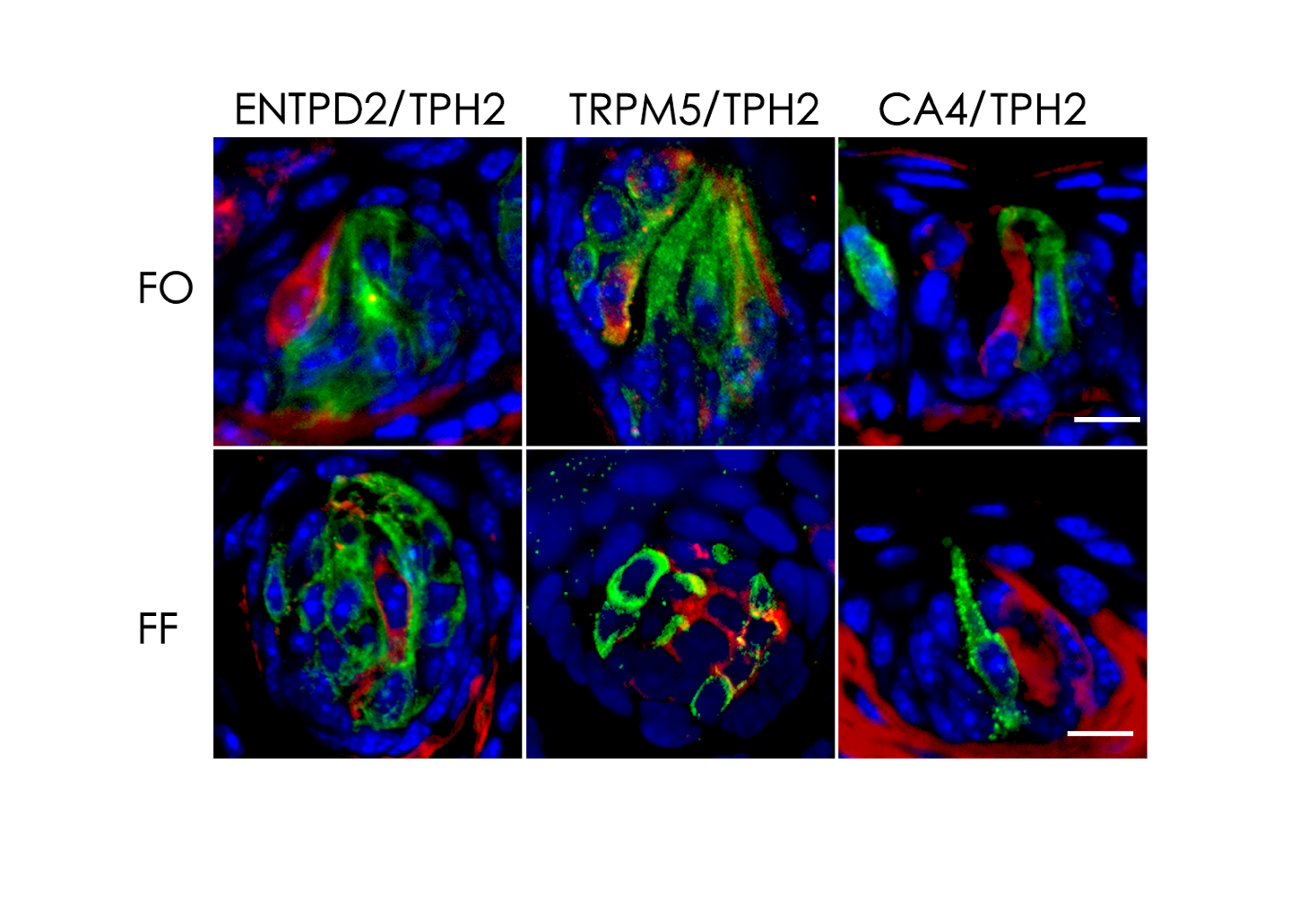


**Supplemental Figure 2 TPH2 expression in mouse fungiform and foliate papillae.** Confocal images showing TPH2 immunostaining signals (red) do not overlap with type I cell marker ENTPD2 (green)(left) or type III cell marker CA4 (green)(right), but partially overlap with type II cell marker TrpM5 (green) (middle), in the foliate (top row) and fungiform (bottom row), respectively. Z-Stack: 8 µm. Scale bars: 10 µm.


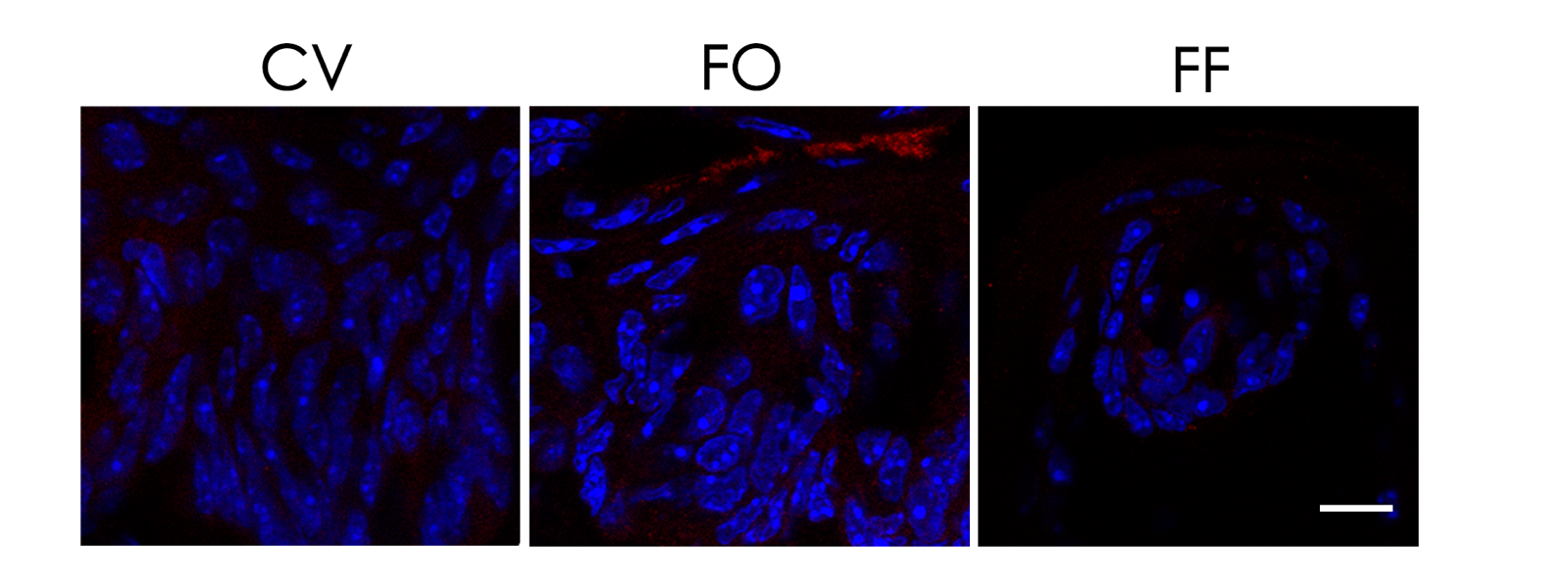


**Supplemental Figure 3 No immunostaining was detected in the negative control tissue sections for the TPH2 antibody.** Confocal images show that omission of the primary antibody against TPH2 resulted in no detectable immunostaining signals in the circumvallate (CV), foliate (FO) and fungiform (FF) taste buds. Z-Stack: 8 µm. Scale bars: 10 µm.


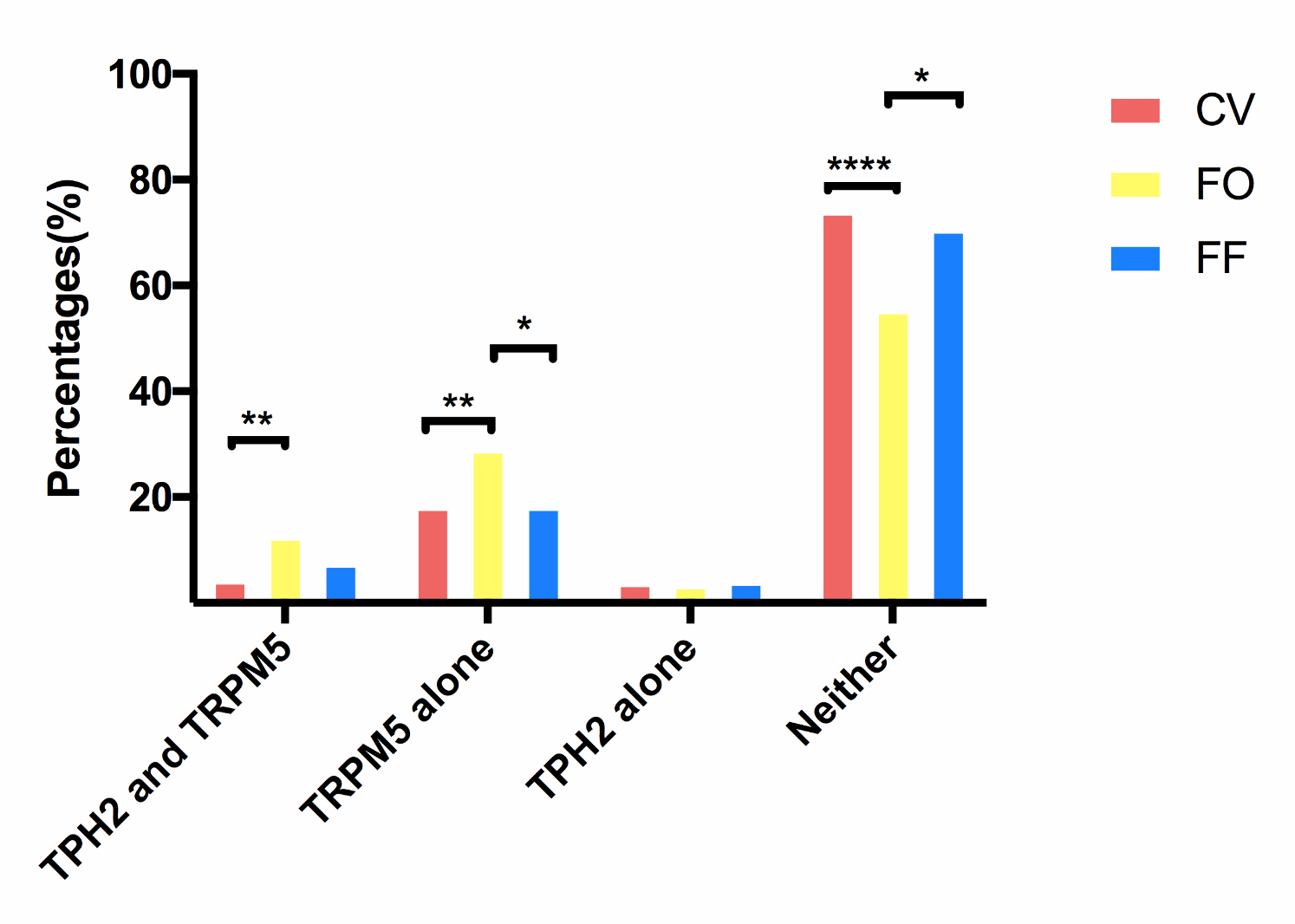


**Supplemental Figure 4 Quantification analysis of taste bud cells expressing TPH2 and TRPM5.** The percentages of taste bud cells expressing both TPH2 and TRPM5, TRPM5 alone, TPH2 alone or neither were calculated and plotted. About 4.2%, 12.4% and 7.4% cells in the circumvallate, foliate and fungiform taste buds, respectively, co-expressed both TPH2 and TRPM5 whereas 18.2%, 28.9% and 18.1% taste bud cells expressed TRPM5 alone, and 3.7%, 3.4% and 3.9% taste bud cells expressed TPH2 alone, in these three types of taste papillae, respectively. About 73.9%, 55.3% and 70.6% of circumvallate, foliate and fungiform taste bud cells expressed neither TPH2 nor TRPM5. Statistical significance was assessed using multiple two-tailed Student’s t-tests (*, P < 0.05; **, P < 0.01; ***, P < 0.001; ****, P < 0.0001).


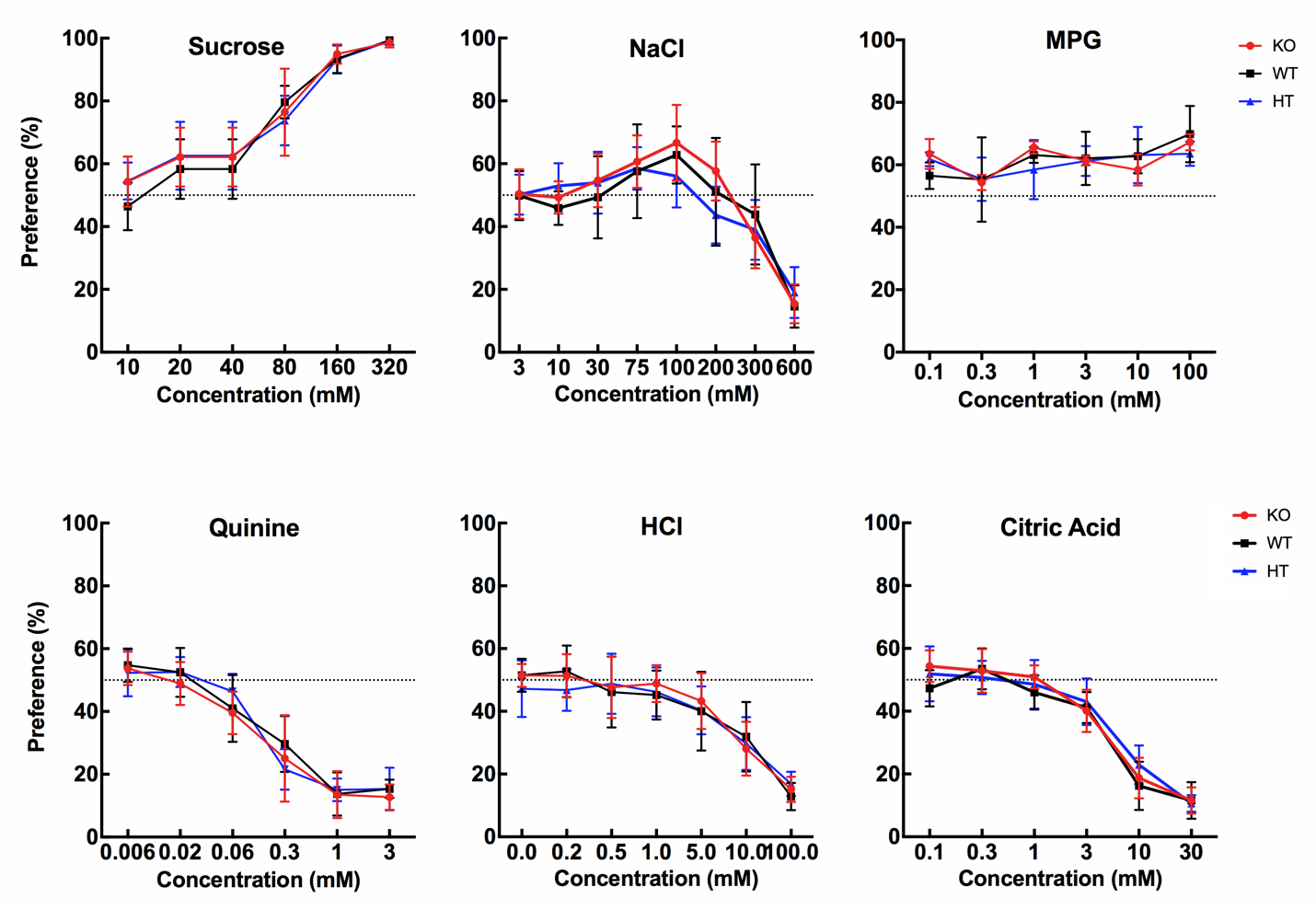


**Supplemental Figure 5 Two-bottle preference tests of *Tph2*-KO, heterozygotes and wild type mice in response to 6 different tastants.** Preference scores of *Tph2*-KO (KO, in red), *Tph2*- heterozygotes (HT, in blue) and wild-type (WT, in black) mice were plotted over the tastant concentrations with a dotted line at 50%. Two-way ANOVA analyses were performed for the 6 tastants: sucrose (F (2, 132) = 1.077, P = 0.3436), NaCl ( F (2, 163) = 0.9704, P = 0.3811), potassium glutamate (MPG; F (2, 37) = 0.1683, P = 0.8457), citric acid (F (2, 107) = 1.459, P = 0.2370), hydrochloric acid (HCl; F (2, 293) = 0.7764, P = 0.4610), quinine sulfate (F (2, 158) = 1.579, P = 0.2094). No significant changes were found between different genotype groups.


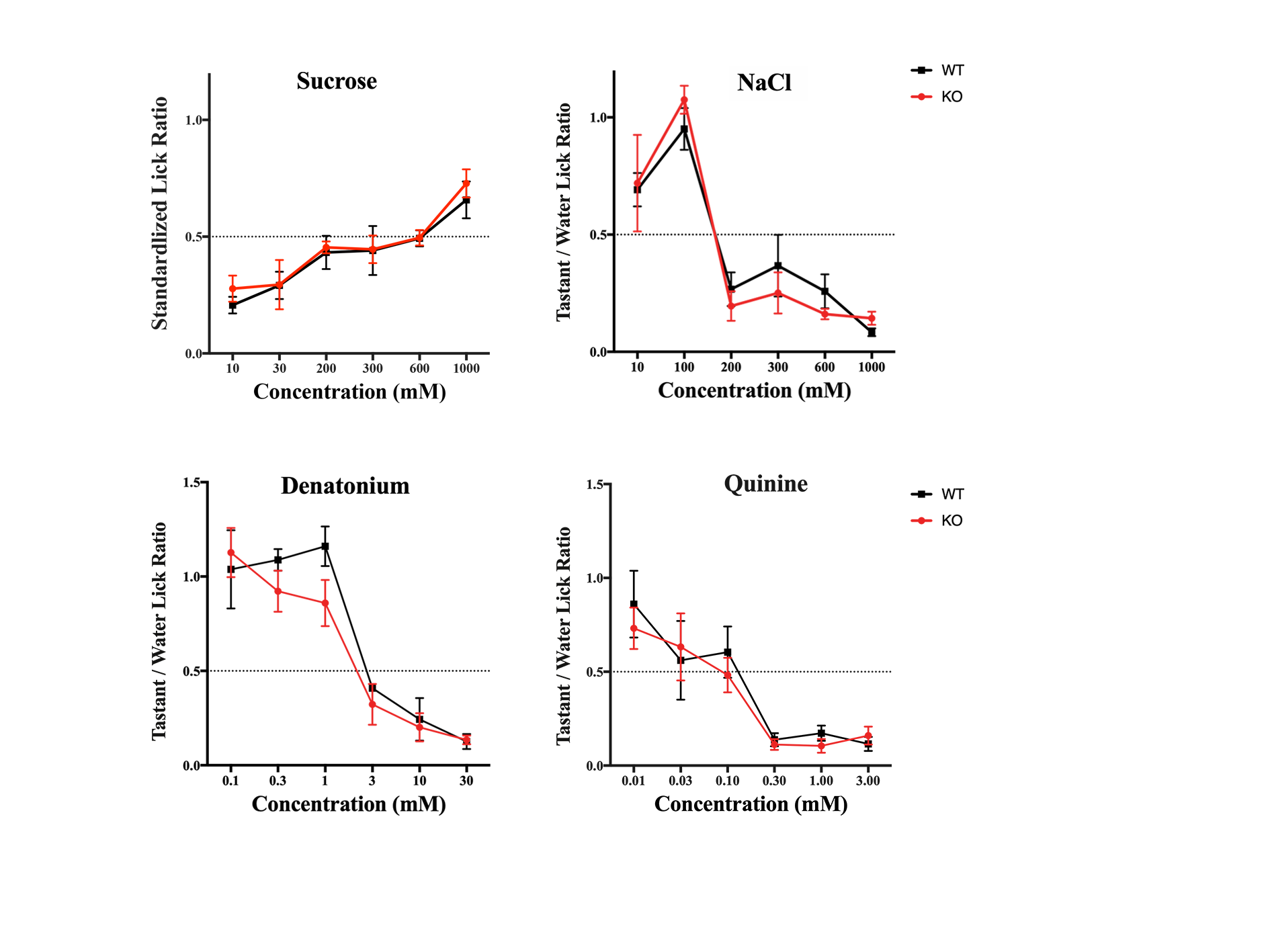


**Supplemental Figure 6 Lickometer tests of *Tph2*-KO and wild type mice in response to different tastants.** The standardized lick ratios were determined for the licking responses to sucrose while the tastant/water lick ratios were used for the responses to all other tastants. The ratio values (means ±S.E.M.) from *Tph2*-KO (KO, in red) and wild-type (WT, in black) mice were plotted over the tastant concentrations with a dotted line at 0.50. Two-way ANOVA analyses were performed for the tastants: sucrose (F (1, 40) = 0.6277, P = 0.4329), NaCl (F (1, 66) = 0.07234, P = 0.7888), denatonium (F (1, 41) =1.78, P = 0.1895), quinine sulfate (F (1, 56) = 0.4421, P = 0.5089). No significant changes were found between different genotype groups.


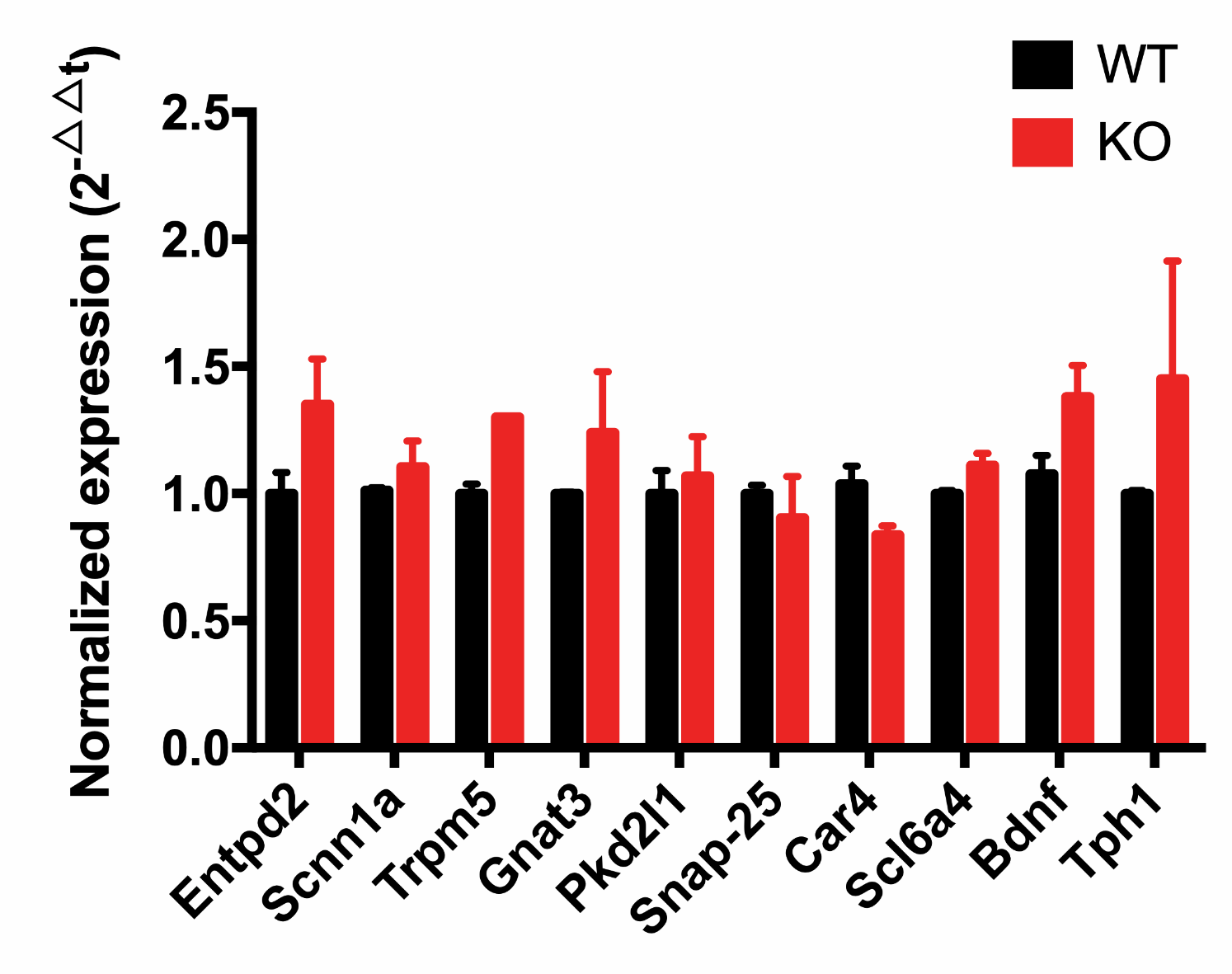


**Supplemental Figure 7 Effect of *Tph2* knockout on expression of some taste-related genes.** RT-qPCR analysis of taste-related genes’ expression in taste epithelium containing circumvallate, fungiform and foliate taste buds of WT (black) versus *Tph2* KO (red) samples.

**Supplemental Figure 2 Quantification analysis of taste bud cells expressing TPH2 and TRPM5.** Numbers of TRPM5-positive cells and TPH2-positive cells from circumvallate, foliate and fungiform sections obtained from C57 mice. The percentage of TPH2-positive cells in the population of TRPM5-positive cells was calculated and plotted.
